# Comprehensive analysis of KLF family reveals KLF6 as a promising prognostic and immune biomarker in pancreatic ductal adenocarcinoma

**DOI:** 10.1186/s12935-024-03369-3

**Published:** 2024-05-21

**Authors:** Jiayu Lin, Pengyi Liu, Keyan Sun, Lingxi Jiang, Yang Liu, Yishu Huang, Jia Liu, Minmin Shi, Jun Zhang, Ting Wang, Baiyong Shen

**Affiliations:** 1 Department of General Surgery, Pancreatic Disease Center, Ruijin Hospital, Shanghai Jiaotong University School of Medicine, Shanghai, China; 2 Shanghai Key Laboratory of Pancreatic Neoplasms Translational Medicine, Shanghai, China; 3 Research Institute of Pancreatic Diseases, Shanghai Jiaotong University School of Medicine, Shanghai, China; 4 State Key Laboratory of Oncogenes and Related Genes, Shanghai Jiaotong University, Shanghai, China; 5 Institute of Translational Medicine, Shanghai Jiaotong University, Shanghai, China; 6 Department of Pathology, Ruijin Hospital Affiliated to Shanghai Jiao Tong University School of Medicine, Shanghai, China; 7 Department of General Surgery, Pancreatic Disease Center, Ruijin Hospital, Shanghai Jiaotong University School of Medicine, Shanghai, People’s Republic of China; 8 Research Institute of Pancreatic Diseases, Shanghai Key Laboratory of Translational Research for Pancreatic Neoplasms, Shanghai Jiaotong University School of Medicine, Shanghai, People’s Republic of China; 9 State Key Laboratory of Oncogenes and Related Genes, Institute of Translational Medicine, Shanghai Jiaotong University, Shanghai, People’s Republic of China

**Keywords:** PDAC, KLFs, TIME, Prognosis, Immune, KLF6

## Abstract

**Background:**

Pancreatic ductal adenocarcinoma (PDAC) is one of the deadliest tumors worldwide, with extremely aggressive and complicated biology. Krüppel-like factors (KLFs) encode a series of transcriptional regulatory proteins and play crucial roles in a variety of processes, including tumor cell differentiation and proliferation. However, the potential biological functions and possible pathways of KLFs in the progression of PDAC remain elusive.

**Methods:**

We systematically evaluated the transcriptional variations and expression patterns of KLFs in pancreatic cancer from the UCSC Xena. Based on difference analysis, the non-negative matrix factorization (NMF) algorithm was utilized to identify the immune characteristics and clinical significance of two different subtypes. The multivariate Cox regression was used to construct the risk model and then explore the differences in tumor immune microenvironment (TIME) and drug sensitivity between high and low groups. Through single-cell RNA sequencing (scRNA-seq) analysis, we screened *KLF6* and further investigated its biological functions in pancreatic cancer and pan-cancer.

**Results:**

The KLFs exhibited differential expression and mutations in the transcriptomic profile of PDAC. According to the expression of KLFs, patients were classified into two distinct subtypes, each exhibiting significant differences in prognosis and TIME. Moreover, the KLF signature was developed using univariate Cox and Lasso regression, which proved to be a reliable and effective prognostic model. Furthermore, the KLF_Score was closely associated with immune infiltration, response to immunotherapy, and drug sensitivity and we screened small molecule compounds targeting prognostic genes separately. Through scRNA-seq analysis, *KLF6* was selected to further demonstrate its role in the malignance of PC in vitro. Finally, pan-cancer analysis emphasized the biological significance of *KLF6* in multiple types of tumors and its clinical utility in assessing cancer prognosis.

**Conclusion:**

This study elucidated the pivotal role of KLF family genes in the malignant development of PC through comprehensive analysis and revealed that *KLF6* would be a novel diagnostic biomolecule marker and potential therapeutic target for PDAC.

**Supplementary Information:**

The online version contains supplementary material available at 10.1186/s12935-024-03369-3.

## Introduction

Pancreatic ductal adenocarcinoma (PDAC) is a common malignant cancer of the digestive tract and has ranked as one of the most lethal tumors [1]. Due to the rapid progression and ease of recurrence or metastasis of the tumor, as well as the absence of early diagnostic biomarkers, the 5-year survival rate for PC patients is less than 10% [2]. Currently, chemotherapy and surgery offer limited efficacy, especially in advanced cases. Thus, to improve the clinical outcomes of patients, identifying sensitive biomarkers for the early diagnosis and effective therapy of PDAC is urgently needed.

The Krüppel-like factors (KLFs) family contains 17 transcription factors, each with three conserved Cys2/His2 zinc finger domains in their C-terminal region [3]. They specifically bind to promoter and enhancer regions, regulating cell activities such as proliferation, apoptosis, differentiation, and embryonic development [4]. Multiple members of KLFs play key roles in mediating numerous signaling pathways to regulate the biological process [5]. Recently, increasing evidence has shown that dysregulated expression of KLFs is an important factor in the progression of several malignant tumors [6]. They could generally act as suppressors or oncogenes in different tumors. In primary hepatocellular carcinoma, loss of *KLF4* promotes oncogenic TGF-β signaling by activating Samd7 to facilitate the malignant progression [7]. Additionally, KLF4 also inhibits the ERK/ JNK/ NF-κB signaling pathway in non-small cell lung cancer [8]. Conversely, KLF5 regulates cell cycle-related genes, promoting the cell cycle and cancer progression, including pancreatic, gastric, and breast cancers [9–11].

Tumor microenvironment (TME) is associated with the malignant progression of tumors, which are filled with various immune cells and stromal components [12]. KLFs also participate in regulating tumor immune microenvironment (TIME). It’s reported that KLF2 enhances B-cell differentiation and function by enhancing the expression of CD62L, CXCR7, and β7-harmonized protein [13]. *KLF5* promotes the transcription of *COX2* and increases the synthesis of PGE2, which binds to EP2 and EP4 receptors on the surface of CD8 + T-cells thereby inhibiting the function of CD8 + T-cells and shaping a suppressive immune microenvironment [14]. In summary, KLFs are crucial for the progression of tumors and the modulation of the immune microenvironment. However, the role of KLFs in PDAC progression remains clearly unelucidated.

In our study, we classified patients with PDAC into two subtypes based on differentially expressed KLFs. The immune microenvironmental differences between subtypes and the prognostic significance were assessed. Using univariate Cox regression and least absolute shrinkage and selection operator (LASSO)-Cox regression analysis, we developed a reliable risk score model and further investigated the associations between immune infiltration, KLF signature, immune checkpoints, and drug sensitivity. Also, the small-molecule drugs were screened by targeting the prognostic genes. Through single-cell RNA sequencing (scRNA-seq), we screened for *KLF6* and explored its biological role in pancreatic cancer cells. Furthermore, we explored the biological and clinical relevance of *KLF6* across different cancer types. Overall, we clarified the expression and prognostic significance of the KLF family in pancreatic cancer and provided new insights for future treatment.

## Materials and methods

### Data preparation and processing

The traditional RNA sequencing (Bulk RNA-seq) of PDAC patients and corresponding clinical characteristics and copy number variation (CNV) data were obtained from the UCSC Xena (https://xenabrowser.net/datapages/). The validation cohort GSE79668 was downloaded from the Gene Expression Omnibus (GEO) database (https://www.ncbi.nlm.nih.gov/geo/) [15]. The scRNA-seq GSE154778 was also gained from the GEO database, consisting of 10 primary tumor tissues and 6 metastatic lesions [16]. We investigated the mutation frequency and patterns of KLF family members using the cBioPortal database (https://www.cbioportal.org/).

### Identification of differentially expressed genes and NMF algorithm analysis

We performed “DESeq2” R package to find differentially expressed genes (DEGs) of KLFs in PDAC. |log2Fold change (FC)|> 1 and *p* < 0.05 were considered as the filter criteria. According to the expression of differentially expressed KLFs, we utilized the “NMF” R package to explore molecular subtypes of patients with PDAC. The optimal number of clusters (k) was selected based on the cophenetic, dispersion, and silhouette metrics. Eventually, patients were divided into the most significant different molecular clusters when k = 2. The survival curve of two clusters was calculated by the “survival” R package. The “pheatmap” R package was used to visualize the differential expression KLFs and clinicopathological characteristics of the two subtypes.

### Gene set variation analysis (GSVA) and Gene set enrichment analysis (GSEA)

The “clusterProfiler” and “GSVA” R packages were applied for functional enrichment analysis. The Kyoto Encyclopedia of Genes and Genomes (KEGG) database was used for GSVA analysis which regarded adjusted *p* < 0.05 as statistically significant enrichment. For the analysis of gene set enrichment, GSEA 4.2.3 was employed. Hallmark gene sets were collected from the MSigDB [17]. A *p* < 0.05 and |normalized enrichment score (NES) |> 1.5 were regarded to indicate significant differences between the two distinct subgroups.

### Immune landscape analysis

To compare the TIME between the different subgroups, we performed the CIBERSORT algorithm to calculate the scores of immune cells in 22 types, which derived the proportion of immune cell infiltration. Additionally, the stromal score, immune score, and estimate score were computed by the "estimate" R package. To predict the immunotherapy response in two subsets, the expression of programmed death-ligand 1 (PD-L1 or CD274), programmed death-1 (PD-1or CD279), and cytotoxic T-lymphocyte-associated protein 4 (CTLA-4) were calculated.

### Construction of the risk score model

Differentially expressed KLFs underwent univariate Cox regression to determine their prognostic relevance, and genes with *p* < 0.05 were selected (Table S1). The "glmnet" R package and the least absolute shrinkage and selection operator (LASSO) regression were used in conjunction to prevent overfitting. The multivariate Cox regression was subsequently utilized to assess the remaining genes, generating a risk score for each patient with PDAC (Table S2). The KLF_score formula was as follows: KLF_score = Σ (Expi * coefi). Patients were separated into high- and low-risk groups based on median values. Prognostic significance and discrimination of the risk score model were evaluated using the "survival" and "timeROC" R packages for Kaplan–Meier (K-M) survival analysis and time-dependent receiver operating characteristic (ROC) curve, respectively. Principal component analysis (PCA) was performed using the R tool "plotly". The TCGA Pancreatic Cancer (PAAD) dataset from UCSC Xena was utilized as the training set to develop the prognostic model, while GSE79668 served as an independent validation set to assess the accuracy and predictive performance of the model.

### TIME analysis and prediction of immunotherapy response

A correlation analysis was used to explore the relationship between immune cell abundance and KLF_score. Additionally, immune processes were examined based on KLF_score, using single-sample gene set enrichment analysis (ssGSEA). Additionally, the effectiveness of immunotherapy was evaluated by calculating the expression of the immune checkpoint-related genes, including *BTLA*, *BTNL2, CD160*, *CD200R1*, *CD244*, *CD27*, *CD274*, *CD276*, *CD28*, *CD40*, *CD40LG*, *CD44*, *CD48*, *CD80*, *CTLA4*, *HHLA2*, *ICOS*, *IDO1*, *IDO2*, *LAG3*, *LGALS9*, *NRP1*, *PDCD1*, *TIGIT*, *TMGD2*, *TNFRSF14*, *TNFRSF18*, *TNFRSF9*, *TNFSF14*, *TNFSF18*, *TNFSF4* and *VTCN1*. The Tumor Immune Dysfunction and Exclusion (TIDE), dysfunction, and tumor mutation burden (TMB) scores were used to predict immunotherapy response high TMB and low TIDE and dysfunction scores indicated a greater response to immunotherapy. The correlation between TMB and KLF_score and the potential of KLF_score to predict the response to immunotherapy were examined.

### Drug sensitivity and screening

We employed the "oncoPredict" R package to assess the therapeutic efficacy variations of targeted therapy between the low- and high-risk categories. The Genomics of Drug Sensitivity in Cancer (GDSC) database was used to find data on drug sensitivity [18]. Additionally, we used Autodock for molecular docking to explore the interactions of small-molecule drugs with prognostic genes. We retrieved the list of drugs interacting with prognostic genes from the Comparative Toxicogenomics Database (CTD), and the structures of the small molecules from the PubChem database [19, 20]. Relevant biological macromolecular structures encoded by the prognostic genes were retrieved from the Uniport database. The molecule that interacted most strongly with the molecular target having the lowest binding energy was identified through automatic standard docking. PyMol was used for visualization.

### Establishment and validation of the predictive nomogram

Clinical parameters (age, gender, etc.) and KLF_score underwent univariate Cox and multivariate Cox regression to build an enhanced prediction model. Both univariate and multivariate Cox regressions yielded independent prognostic factors with *p* < 0.05. We used the "regplot" R package to create an interactive nomogram that predicted the 1-, 3-, and 5-year overall survival. Calibration curves gauged the precision of the prediction, while K-M survival analysis and time-dependent ROC curves assessed relevance and discrimination for the 1-, 3-, and 5-year overall survival. Model accuracy was confirmed by the validation dataset GSE79668.

### ScRNA-seq analysis

GSE154778 was performed using the Seurat (v4.1.1) in R. The primary processes in the analysis pipeline include object construction, data normalization, data downscaling, clustering, and marker gene identification. Cell-type annotation utilized a standard Seurat process, referencing a prior study and the CellMarker database [21].

### Pan-cancer analysis

To further investigate the biological importance of *KLF6* in various cancers, we assessed its differential expression in pan-cancer. We also analyzed its clinical prognostic value and its association with TMB and microsatellite instability (MSI). Additionally, the co-expression analysis was performed to investigate the association between *KLF6* and immunostimulators and immunoinhibitors.

### Clinical samples collection

20 pairs of samples from patients with PDAC were collected from Ruijin Hospital affiliated to School of Medicine, Shanghai Jiaotong University. The procedure was approved by the Medical Ethics Committee of the hospital. Before collecting the samples, we obtained informed consent from each patient. Each sample contained a tumor and adjacent normal tissue. All patients had histopathologic confirmation of PDAC and had never received chemotherapy. Samples were used for RNA extraction.

### Cell culture and transfection

Pancreatic cancer cell lines (PATU-8988, PANC-1, hTERT-HPNE, ASPC-1, and CAPAN-1) were purchased from the Cell Bank of the Chinese Academy of Sciences (Shanghai, China). They were cultured in RMPI 1640 with 10% fetal bovine serum and 1% Penicillin streptomycin. Short hairpin RNAs (shRNA) targeting *KLF6* were mixed with HilyMax (Dojindo) for 15 min, and the mixture was added to the supernatant to achieve transient transfection. All cells were cultured at 37 °C and 95% humidity, in a 5% CO_2_ atmosphere. The protein expression level was assessed 3 days later.

### Western blotting

Cells were lysed using RIPA buffer (Abclonal, Wuhan, China) containing 1% protease and phosphatase inhibitors (MCN Biotech). The mixture was set on ice for 10 min and heated to 100 °C for 30 min. The obtained proteins were separated using 10% sodium dodecyl-sulfate polyacrylamide gel electrophoresis, transferred to polyvinylidene fluoride membranes, and blocked. The membranes were incubated overnight at 4 °C with primary antibodies, including anti-KLF6 (abclonal, A10011) and anti-β-Actin (abclonal, AC004) antibodies. The next day, the membranes were incubated with a secondary antibody. An enhanced chemiluminescence (ECL) detection system was used for the visualization of the proteins.

### Cell proliferation assay

Cell activity was measured using Cell Counting Kit-8 (CCK-8) (Meilunbio, China), according to the manufacturer’s instructions. Transfected cells (2000/well) were seeded in 96-well plates. Prior to the assay, 90 μL complete RMPI 1640 and 10 μL CCK-8 were added to each well and incubated at 37 °C for 2 h. Absorbance at 450 nm was measured, representing cell activity.

For the colony formation assay, 1000 tumor cells or transfected cells were cultured in 6-well plates for 10 days. On day 10, the cell supernatant was discarded, and cells were stained with a crystal violet staining solution and subsequently imaged.

### Patient-derived organoid (PDO) construction

According to the instruction of the human Tumor Dissociation Kit (Miltenyi), organoids were extracted and digested from tumor tissues of PDAC patients who had not received chemotherapy at Ruijin Hospital. Organoids were seeded into the Matrigel (Corning) and then cultured in OmaStem^®^ Pan-cancer Advanced (OmaStem). After transfecting the plasmids, organoids were digested by TrypLETM Express (ThermoFisher) and seeded in 96-well plates. Thereafter, representative images were obtained every 5 days, and proliferative activity was assayed by CellTiter-Glo^®^ 3D Cell Viability Assay (Promega).

### Transwell assay

For migration, treated and control cells were suspended in 200 μl serum-free DMEM in the upper chamber with 2 × 10^4^ cells per well. As for invasion, the upper portion of plates was covered with Matrigel (Corning, NY, USA). In addition,700 μl complete RMPI 1640 in the lower chamber was available. After 48 h, cells passing through membranes were fixed with 4% PFA stained with 1% crystal violet and counted under the microscope.

### RNA extraction and Quantitative Real-Time PCR (qRT-PCR)

Total RNA was extracted using TRIzol reagent (Invitrogen, USA). HiScript Reverse Transcriptase (Vazyme, China) was used to conduct reverse transcription. SYBRGreen PCR Kit (Vazyme, China) was used for RT-PCR, with β-actin as an internal control. The following primer pairs were used:

*KLF6*: GGCAACAGACCTGCCTAGAG (Forward Sequence).

*KLF6*: CTCCCGAGCCAGAATGATTTT (Reverse Sequence).

*β-Actin*: GTCATTCCAAATATGAGATGCGT (Forward Sequence).

*β-Actin*: GCTATCACCTCCCCTGTGTG (Reverse Sequence).

### Immunohistochemistry (IHC)

The tissues underwent paraffin embedding, formalin fixation, and sectioning onto slides. After that, the conventional streptavidin–biotin-peroxidase complex method was used for IHC staining. The slides were deparaffinized and rehydrated. Subsequently, they underwent antigen retrieval, inactivation, primary and secondary antibody incubation, DAB staining, and sealing. Ultimately, representative images were taken under a microscope, and the area and intensity of staining were used to calculate IHC scores.

### Statistical analysis

All statistical tests and analyses were conducted using GraphPad Prism 7 and R (version 4.1.1). Clustering analysis was performed using the "NMF" R package. Survival analysis employed the Kaplan–Meier (KM) method and log-rank tests from the "survival" R package. The prognosis was assessed using the area under the ROC curve (AUC) via the "timeROC" R program. Correlation analysis was performed using the Spearman correlation test. The Student’s two-tailed unpaired t-test and one-way ANOVA were used for statistical analyses. Statistical significance was set as *P* < 0.05.

## Results

### Identification of differently expressed KLFs in PDAC

**Fig. 1:**
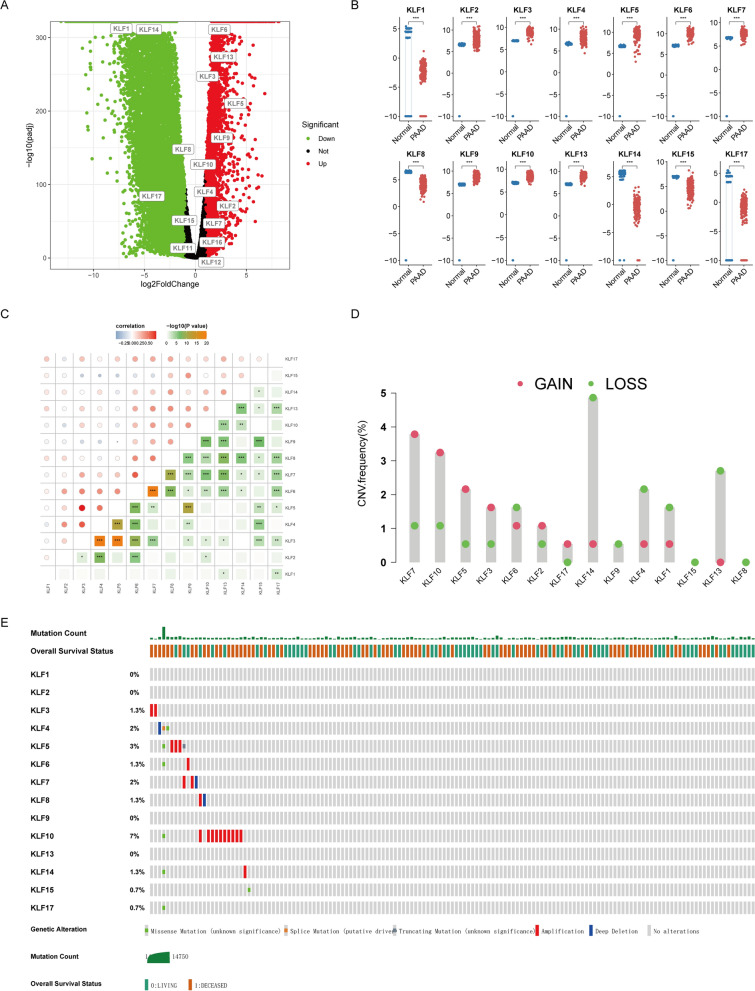
Assessment of KLF family expression and genetic variations in PDAC. **A**, **B** The volcano gram and box plots showed differentially expressed KLFs in tumor tissues and adjacent normal tissues **C** Expression correlation analysis of KLFs **D** The CNV frequency of KLFs in PDAC. The frequency of the changes was indicated by the height of the bars. The green dot denoted CNV deletions (loss), whereas pink indicated CNV amplification (gain). **E** Mutation frequency and type of KLFs. (*p < 0.05; **p < 0.01; ***p < 0.001; ns no significance)

To elucidate the transcriptome profiles of the KLF family in pancreatic cancer, we accessed mRNA expression data from the UCSC Xena database. Using | log2FC |> 1 and *p*-value < 0.05 as criteria for differential expression, we identified significant expression differences among most KLFs except for *KLF11*, *KLF12*, and *KLF16* (Fig. 1A, B). Spearman analysis revealed strong correlations within the KLF family, with the strongest correlation being detected between *KLF3* and *KLF5*, which indicated potential synergistic interactions among KLF family members (Fig. 1C). Next, to explore the genetic variation of KLFs in PDAC, we evaluated CNV and somatic mutation. As shown in Fig. 1D, some KLFs, including *KLF7*, *KLF10*, *KLF5*, *KLF3*, *KLF2*, and *KLF17* exhibited increased CNV, while *KLF6*, *KLF14*, *KLF9*, *KLF4*, *KLF1*, *KLF15*, *KLF13*, and *KLF8* displayed decreased CNV. Notably, we found higher mutation frequencies in *KLF10*, *KLF5*, *KLF4*, *KLF7*, *KLF8*, *KLF3*, *KLF6*, *KLF14*, *KLF15*, and *KLF17* among patients with PDAC (Fig. 1E). The above results illustrated the robust connections between the transcriptomic landscape and the mRNA expression level of KLFs.

### NMF clustering identifying the molecular subtypes of KLFs

**Fig. 2:**
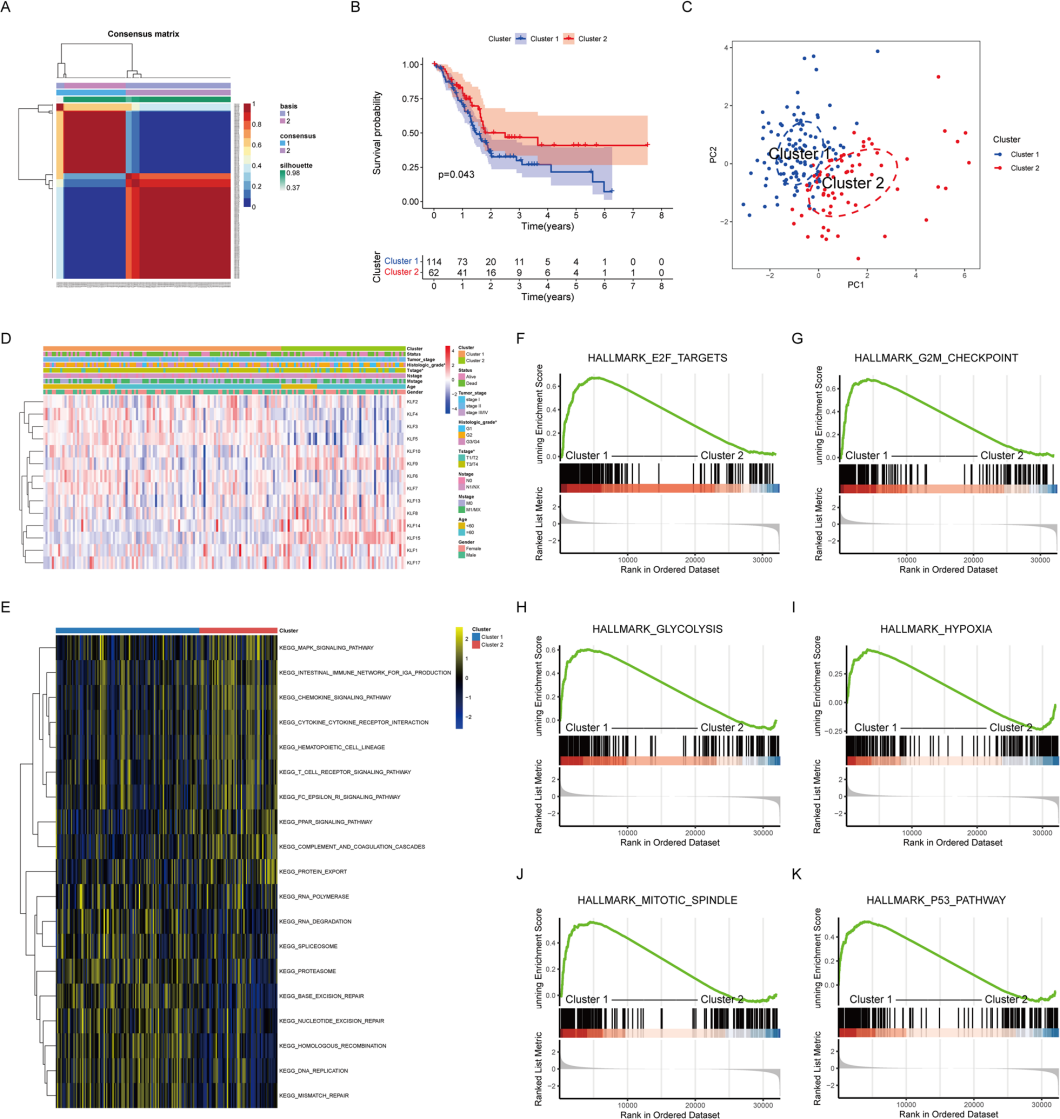
Classifying pancreatic cancer patients based on differentially expressed KLFs. **A** The non-negative matrix decomposition algorithm classified patients into two distinct subgroups (C1 and C2). **B** Kaplan–Meier plots compared the overall survival of the two subgroups (log-rank *p* = 0.043). **C**The two subgroups were clearly distinguished on PCA analysis. **D**The heatmap displayed the expression of KLFs in clusters 1 and 2, as well as the associations between clinicopathological characteristics and different subtypes. **E**The enrichment analysis between cluster 1 and cluster 2. Higher pathway richness was indicated by a yellow pathway, whereas lower pathway enrichment was indicated by a blue pathway. Statistical significance is defined as an adjusted* p* < 0.05. **F**–**K** GSEA revealed the enriched pathways in patients with different subtypes

Based on the differentially expressed KLFs, NMF clustering was performed and the optimal number of clusters was 2 (Fig. S1A,B). Hence, we separated patients into two clusters (Fig. 2A). It could be observed that the prognosis of patients in cluster 1 was worse than that of cluster 2 patients following the survival curve (Fig. 2B). PCA analysis indicated consistent and stable subtypes in the distribution of cluster 1 and cluster 2 (Fig. 2C). The heatmap illustrated the associations between KLF expression patterns within the clusters and their corresponding clinicopathological features (Fig. 2D). To demonstrate the potential underlying biological pathways, GSVA and GSEA were performed. As shown in Fig. 2E, cluster 1 was notably enriched in KEGG_RNA_POLYMERASE, KEGG_PROTEASOME, and KEGG_DNA_REPLICATION, while cluster 2 was markedly enriched in immune-related pathways, such as KEGG_CHEMOKINE_SIGNALING_PATHWAY and KEGG_T_CELL_RECEPTOR_SIGNALING_PATHWAY. Furthermore, GSEA indicated that cluster 1 was influenced by HALLMARK_GLYCOLYSIS, HALLMARK_HYPOXIA, HALLMARK_P53_PATHWAY, and other pathways (Fig. 2F–K). These findings revealed a distinct pattern, indicating that patients in cluster 1 exhibited heightened activation of tumor-related pathways, whereas patients in cluster 2 demonstrated greater activation of immune-related pathways. Consequently, it was deduced that cluster 1 displayed a more pronounced pro-tumorigenic activity, whereas cluster 2 exhibited enhanced immune activity. This disparity in pathway activation plausibly accounted for the divergent prognoses observed between these two groups.

### TIME analysis of molecular subtypes

**Fig. 3:**
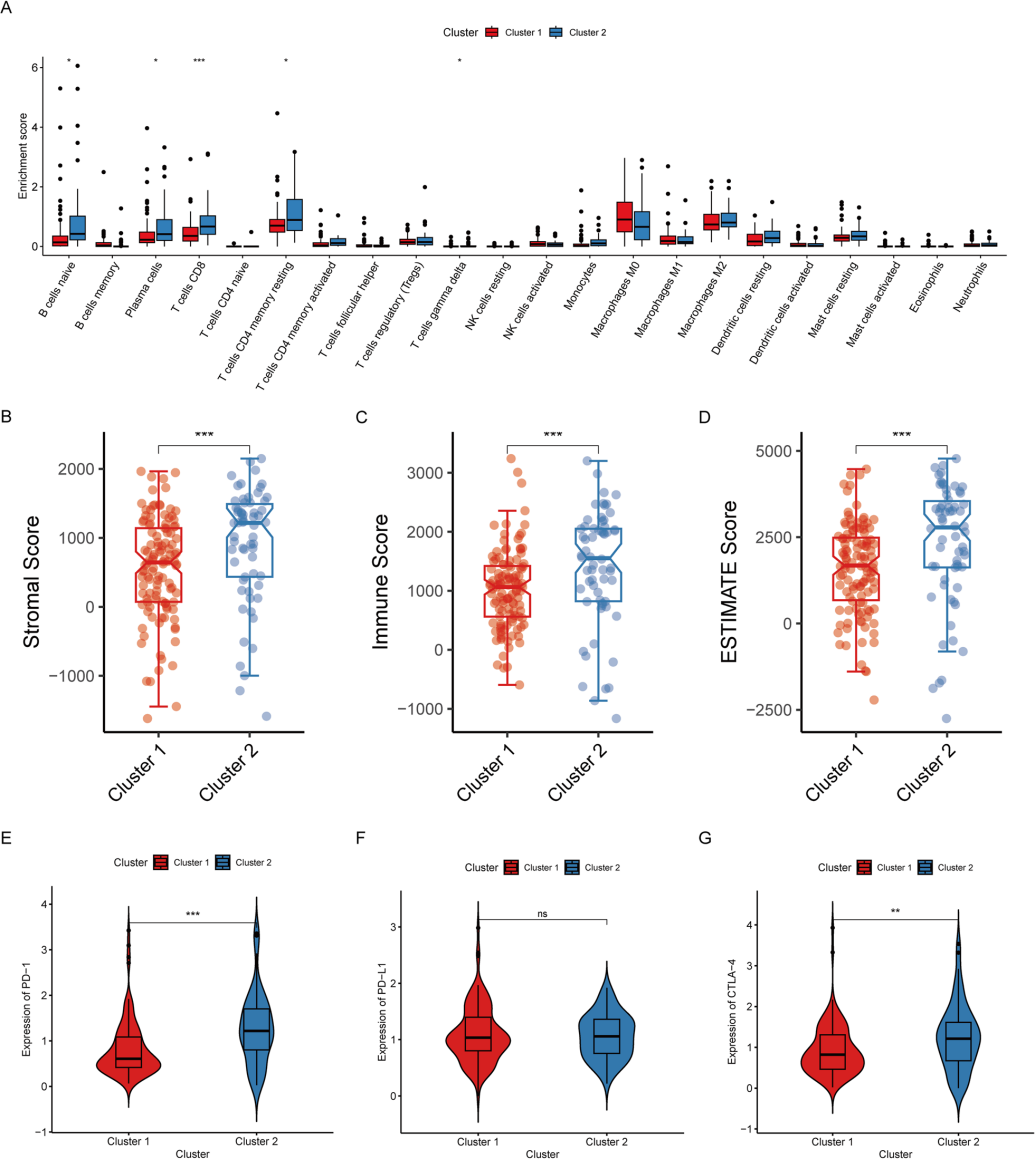
Immune landscape of patients with PDAC in different subgroups. **A** The CIBERSORT algorithm calculated the abundance of each immune infiltrating cell in cluster 1 and cluster 2. **B**–**D** ESTIMATE algorithm evaluated stromal score, immune score, and ESTIMATE score. **E**–**G** Expression levels of immune checkpoints in cluster 1 and cluster 2. (*p < 0.05; **p < 0.01; ***p < 0.001; ns no significance)

As a crucial factor in mediating tumor malignant progression, TIME is implicated in tumor metastasis, immune escape, recurrence, and other processe [22]. Therefore, we hypothesized that there were distinct immunological characteristics between the two subtypes. To confirm this hypothesis, immune infiltration was assessed, and the results revealed that significant enrichment of B cells naïve, T cells CD8, and T cells CD4 memory resting in cluster 2 (Fig. 3A). Furthermore, higher immune score, stromal score, and ESTIMATE score were represented in cluster 2, implying patients in cluster 2 had a more robust immune response and a lower purity of tumor cells in TIME (Fig. 3B–D). Given the pivotal role of the immune checkpoint in tumor immunotherapy, we found that PD-1 and CTLA-4 had significantly higher levels of expression in cluster 2 compared to cluster 1 (Fig. 3E–G). In short, these findings revealed that patients in cluster 2 exhibited immune activation traits, while patients in cluster 1 displayed immunosuppressive features, which suggested that the KLF pattern could better guide patient subtyping and immunotherapy in PDAC.

### Construction and validation of the prognostic model

**Fig. 4:**
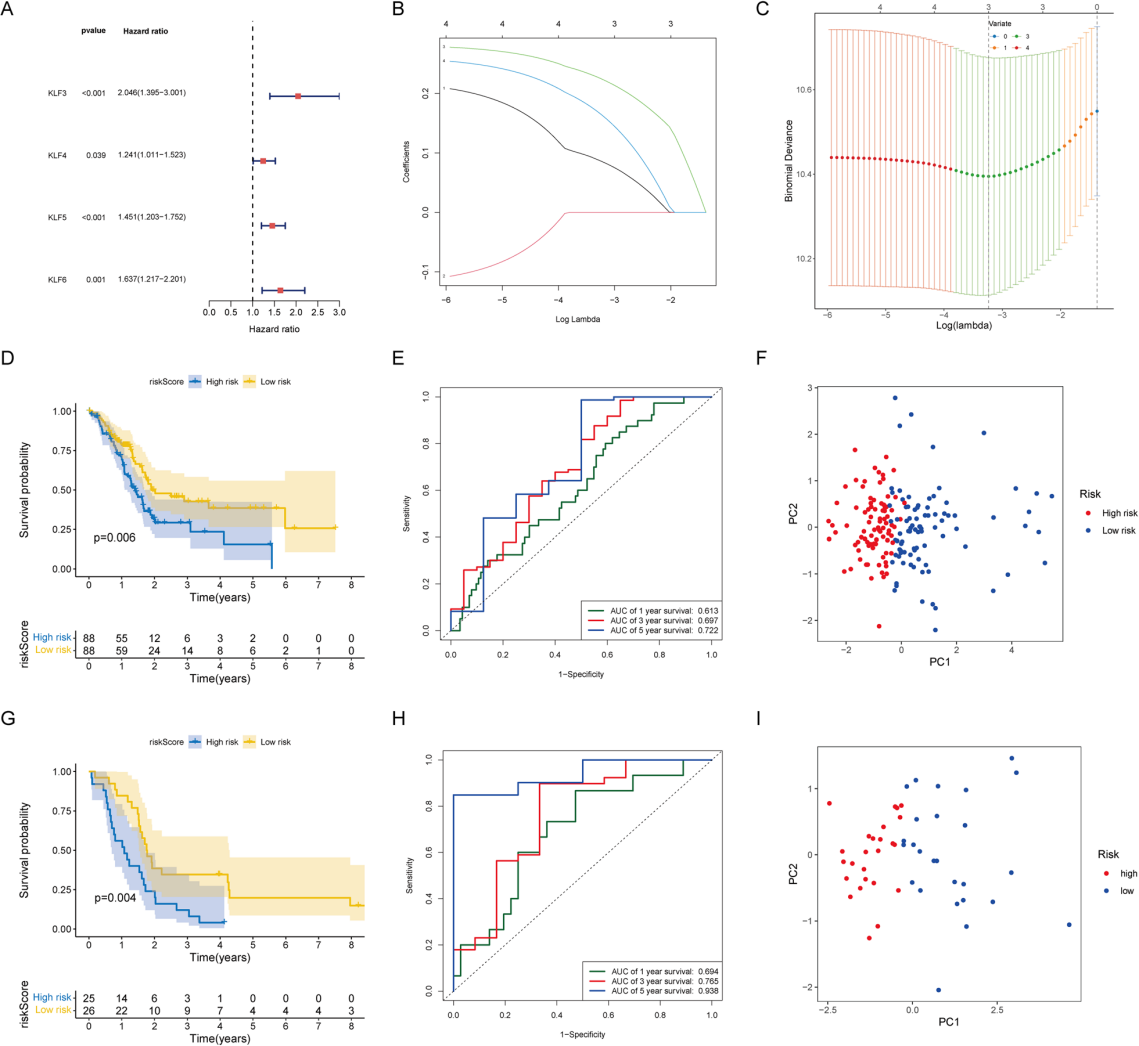
Establishment and verification of KLF signature. **A** Forest plot demonstrated survival-associated KLFs screened by univariate Cox regression analysis. **B** LASSO regression was performed to obtain the prognostic genes. **C** Optimal lambda (λ) was selected based on the cross-validation error curve. **D** Kaplan–Meier survival analysis in the training cohort. **E** The time-dependent ROC curve in the training cohort. **F** PCA analysis in the training cohort. **G** Survival analysis in the validation cohort. **H** The time-dependent ROC curve in the validation cohort. **I** PCA analysis in the validation cohort

To further explore the relationship between KLFs and the prognosis of patients with pancreatic cancer, univariate Cox regression was performed, and we identified 4 significantly prognostic genes (*KLF3*, *KLF4*, *KLF5*, *KLF6*) with *p* < 0.05 (Fig. 4A). Furthermore, we utilized the LASSO-Cox regression to establish a model (including *KLF3*, *KLF5*, and *KLF6*) named the KLF_score model (Fig. 4B, C). The model assigned each patient with PDAC a risk score using the following formula: (0.129498515* expression of *KLF3* + 0.27195101* expression of *KLF5* + 0.246696073* expression of *KLF6*). Patients were classified into high- and low-risk groups based on their median risk score. In both the training and validation sets, patients with high-risk scores had significantly shorter overall survival (Fig. 4D, G). Time-dependent ROC curves demonstrated the model's robustness in the training cohort (5-year AUC, 0.722; 3-year AUC, 0.697; 1-year AUC, 0.613; Fig. 4E) and validation cohort (5-year AUC, 0.938; 3-year AUC, 0.765; 1-year AUC, 0.694; Fig. 4H). PCA and t-SNE effectively differentiated the two risk subgroups (Fig. 4F, I), indicating a clear separation of patients into distinct parts (low- or high-risk) with high discriminatory power.

### The biological contribution of KLF_score

To better understand the biological functions of the two risk groups, GSVA was performed. As shown in Fig S2A, the high-risk group exhibited significant activation of tumor-associated pathways, including the p53 pathway and phospholipid metabolism, while these pathways were less expressed in the low-risk group. Moreover, GSEA confirmed the activation of tumor-associated pathways in the high-risk group (Fig. S2B-G). These results collectively indicated an intimate link between KLF_score and tumor metabolism and proliferation. A higher score indicated heightened tumor activity, which could better explain the worse prognosis observed in the high-risk group.

### Immune characteristics in relation to KLF_score

**Fig. 5:**
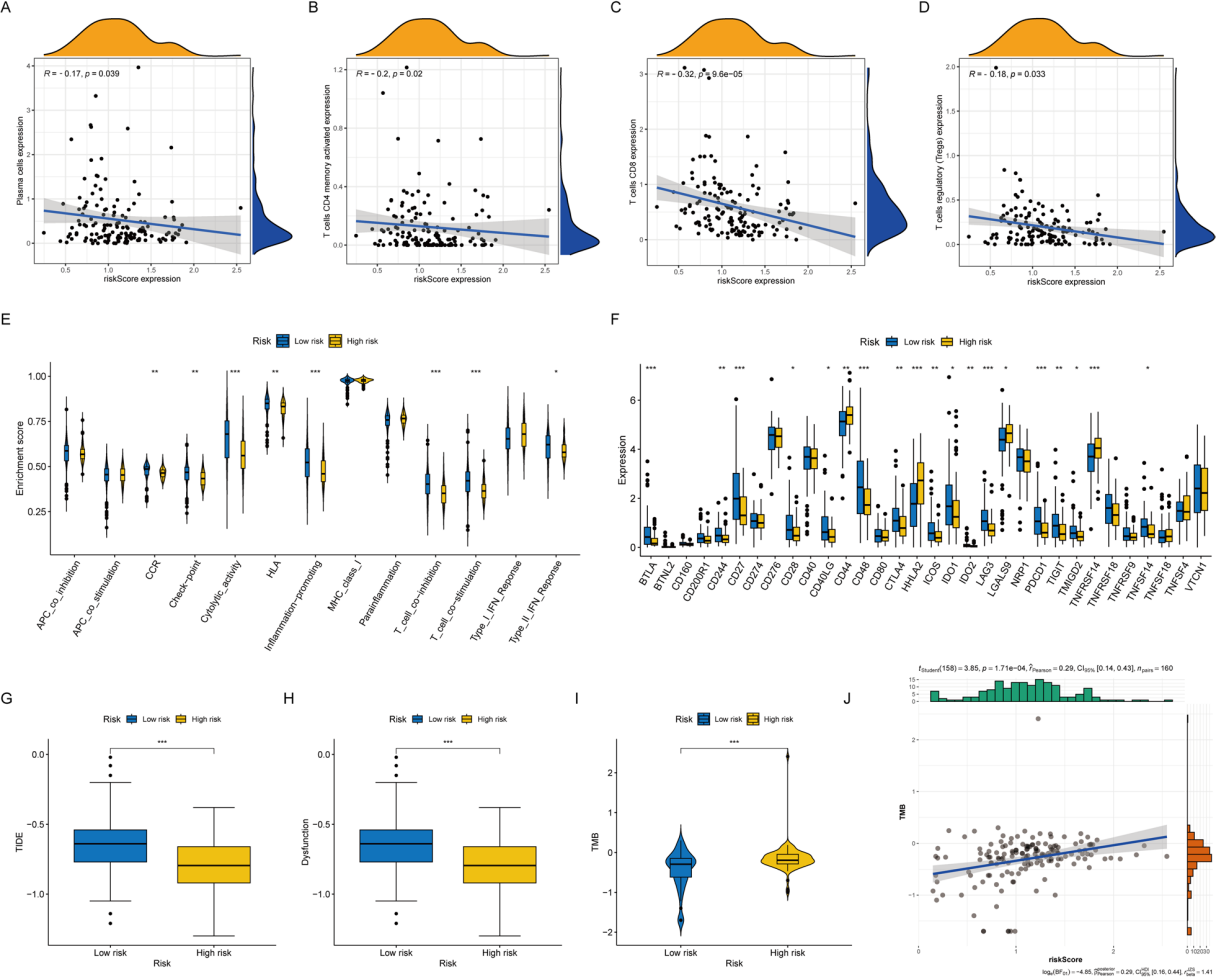
Correlation analysis between KLF_score and tumor immune microenvironment. **A**–**D** Correlation of risk score and immune cells. **E** The boxplot showed the different immune-related functions in the high-risk group and low-risk group. **F** The expression of immune checkpoints between high-risk group and low-risk group. **G**–**I** TIDE score, dysfunction score, and TMB score of high- and low-risk groups. **J** Correlation of risk score and TMB. (*p < 0.05; **p < 0.01; ***p < 0.001; ns no significance)

A mesenchymal space of immune cells exists within pancreatic cancer, including immune cells, extracellular matrix, fibroblasts, and growth factors, which together constitute a suppressive TIME [23]. TIME plays an important role in tumor progression and chemoresistance in PDAC [24]. Thus, we explored potential differences in the TIME across risk groups. Through correlation analysis and assessment of immune processes, we found an inverse relationship between KLF_score and immune cell abundance, suggesting that a lower KLF_score presented a higher immune activity and more activated immune functions (Fig. 5A–E). In other words, the patients in the high-risk group were suffering from an immunosuppressive status. Additionally, the expressions of immune checkpoints were examined, and the results revealed that *CD44*, *TNFRSF14*, *HHLA2*, and *LGALS9* were highly expressed in the high-risk group (Fig. 5F). Moreover, as seen in Fig. 5G–J, the patients in the high-risk group displayed lower TIDE and dysfunction scores and a higher TMB score, which indicated they would be better applied immunotherapy to release immunosuppression and restore immune activity.

### Drug sensitivity prediction and small-molecule drug screening

**Fig. 6:**
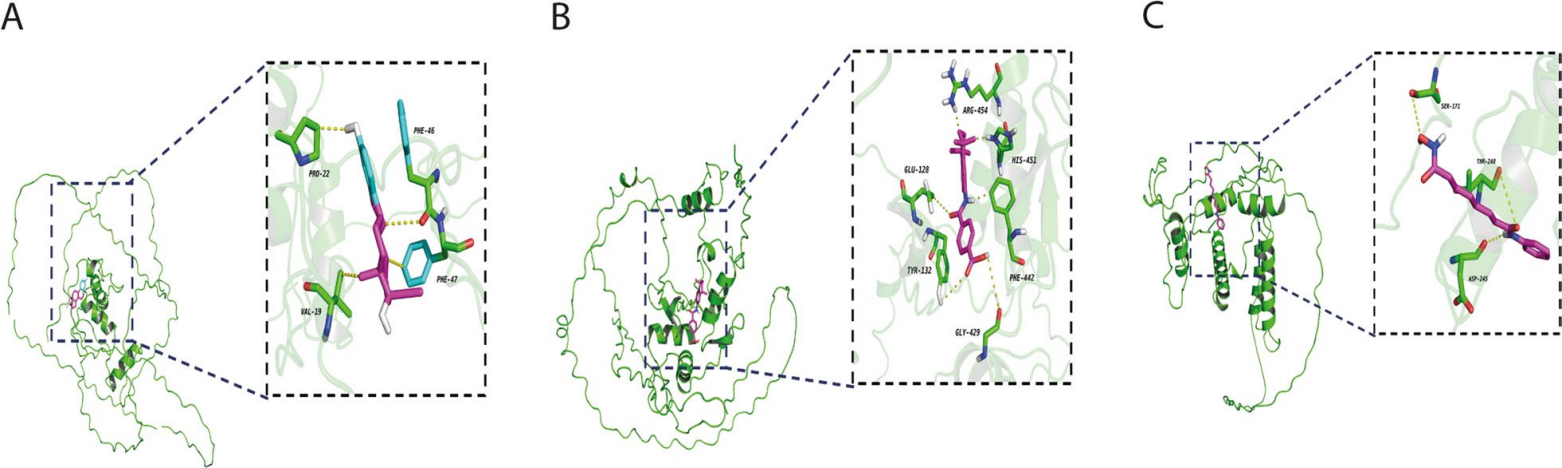
Small molecule drug screening. **A**–**C** 3D structure drawings of molecular docking for *KLF3*-Estradiol, *KLF5*-Tamibarotene and *KLF6*-Vorinostat

Previous study has shown that patients eventually obtained longer survival time when treated by combining chemotherapy with immunotherapy or small molecule targeted drugs [25]. To better guide clinical treatment, we used “OncoPredict” to predict the drug sensitivity of PDAC patients and observed the marked difference between the high-risk and low-risk groups. Patients with low KLF_score were sensitive to gefitinib, bosutinib, cytarabine, nilotinib, pazopanib, sunitinib, and axitinib, while the high-risk group responded better to bleomycin and paclitaxel, which highlighted the relevance of KLF_score to drug sensitivity (Fig. S3A-L). Additionally, we screened the CTD and employed molecular docking and a pharmacological toxicology study to identify three small-molecule drugs targeting prognostic genes. As seen in Fig. 6A, estradiol exhibited a strong binding energy of -7.6 (kcal/mol) with *KLF3*, potentially suppressing its transcription. Vorinostat was better at lowering *KLF6* mRNA expression levels and presented an optimal binding energy of -6.5 (kcal/mol) (Fig. 6B). Tamibarotene exhibited a high binding energy of -8.4 (kcal/mol), indicating potential in inhibiting *KLF5* mRNA expression (Fig. 6C).

### Construction of a nomogram and evaluation of the prognostic model

**Fig. 7:**
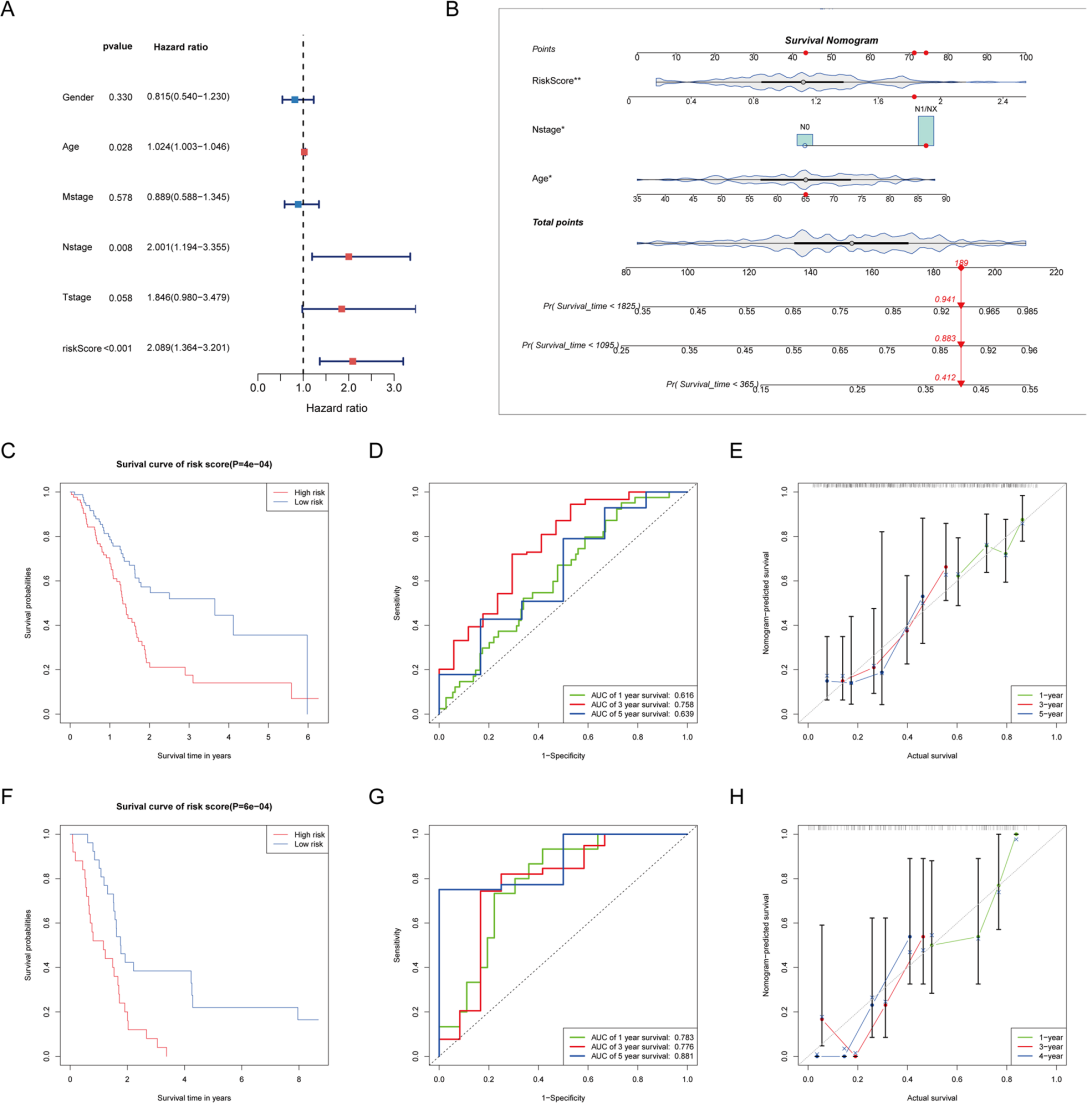
Establishment and evaluation of survival prediction nomogram. **A** Multivariate Cox regression analysis between the risk score and clinical characteristics. **B** A nomogram was established to predict 1-, 3-, and 5-year overall survival based on risk score and clinical features. **C** The comparison of overall survival between high- and low-risk groups in K-M plots. **D** Time-dependent ROC curves for predicting 1-, 3- and 5-year overall survival according to KLF_score. **E** Calibration curves implied the accuracy of 1-, 3-, and 5-year survival. **F**–**H** The results of K-M plots, ROC curves, and calibration curves in the validation cohort

Clinical characteristics are inseparably related to the patient’s prognosis [26]. To determine whether the risk score and other clinical parameters can independently predict pancreatic cancer prognosis, we conducted univariate and multifactor Cox regression and uncovered that age, N stage, and risk score are independent prognostic factors (Fig. 7A). According to the above findings, an interactive nomogram was performed for predicting 1-, 3-, and 5-year overall survival (Fig. 7B). As shown in Fig. 7C, D, the survival of high- and low-risk groups displayed significant differences, with AUC values of 0.616, 0.758, and 0.639 at 1, 3, and 5 years, respectively. Calibration curves confirmed the accuracy and reliability of the model (Fig. 7E). In the validation set, the K-M curve also indicated significantly improved overall survival in the low-risk group, aligning with the training set result (Fig. 7F). Consistent ROC and calibration curves further confirmed the reliability and stability of the prediction model (Fig. 7G, H).

### ScRNA-seq dataset analysis of key genes

**Fig. 8:**
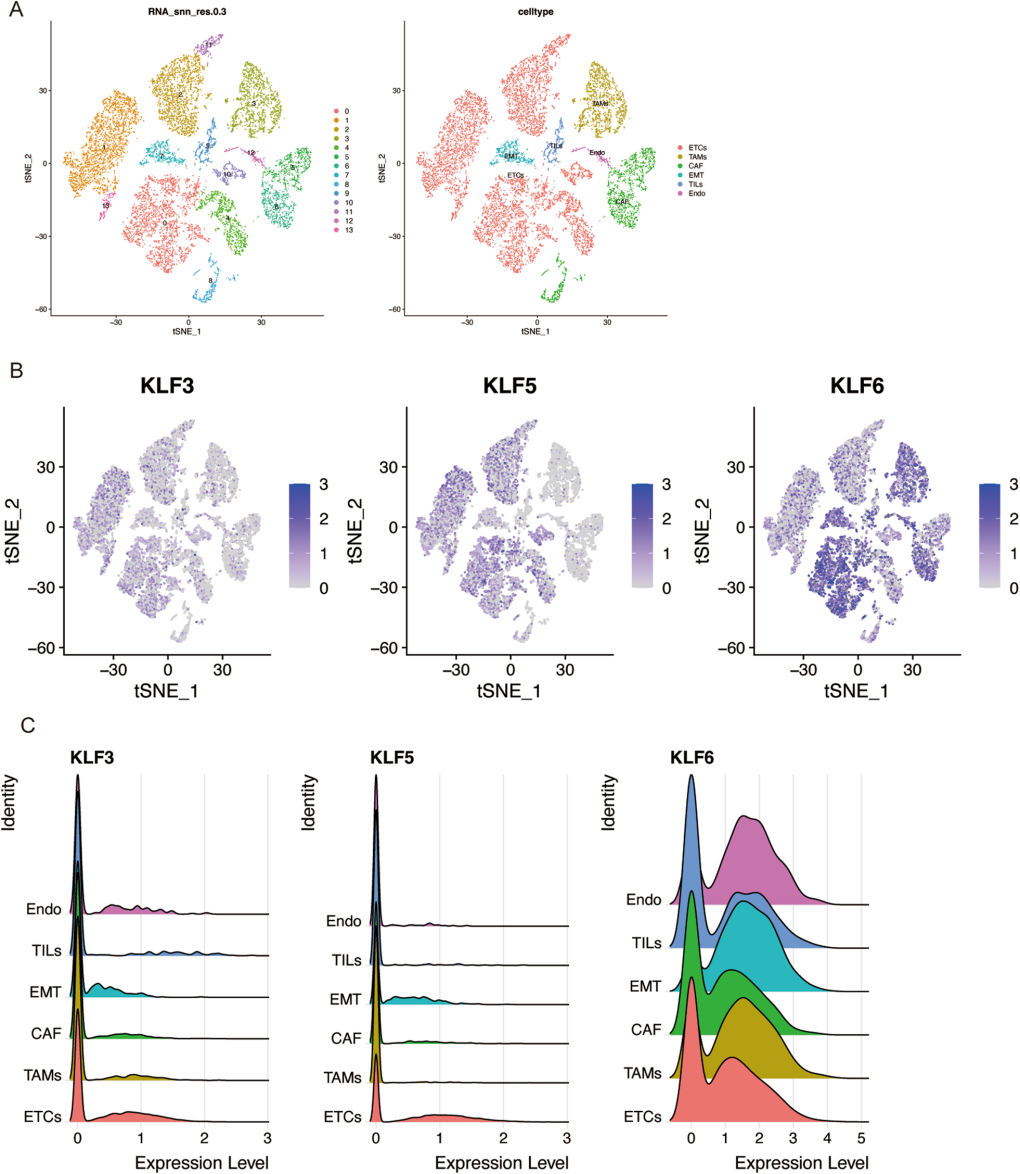
ScRNA-seq analysis for screening key genes. **A** The result of t-SNE dimensionality reduction and cell clusters annotation. **B**–**C** The expression and distribution of *KLF3*, *KLF5*, and *KLF6* in different cell types by Featureplot and ridge map visualization

To further determine the crucial gene of KLFs in the PDAC, we obtained the GSE154778 dataset from GEO and conducted a single-cell analysis. Cell grouping and annotation were performed in a descending manner based on t-SNE (Fig. 8A). As described in Fig. 8B, C, *KLF6* exhibited significantly higher expression levels than *KLF3* and *KLF5* in epithelial tumor cells (ETCs). Moreover, it could be observed that *KLF6* was highly expressed in other immune cells, suggesting it may affect PDAC progression in multiple aspects.

### Biological functions of KLF6 in PDAC

**Fig. 9:**
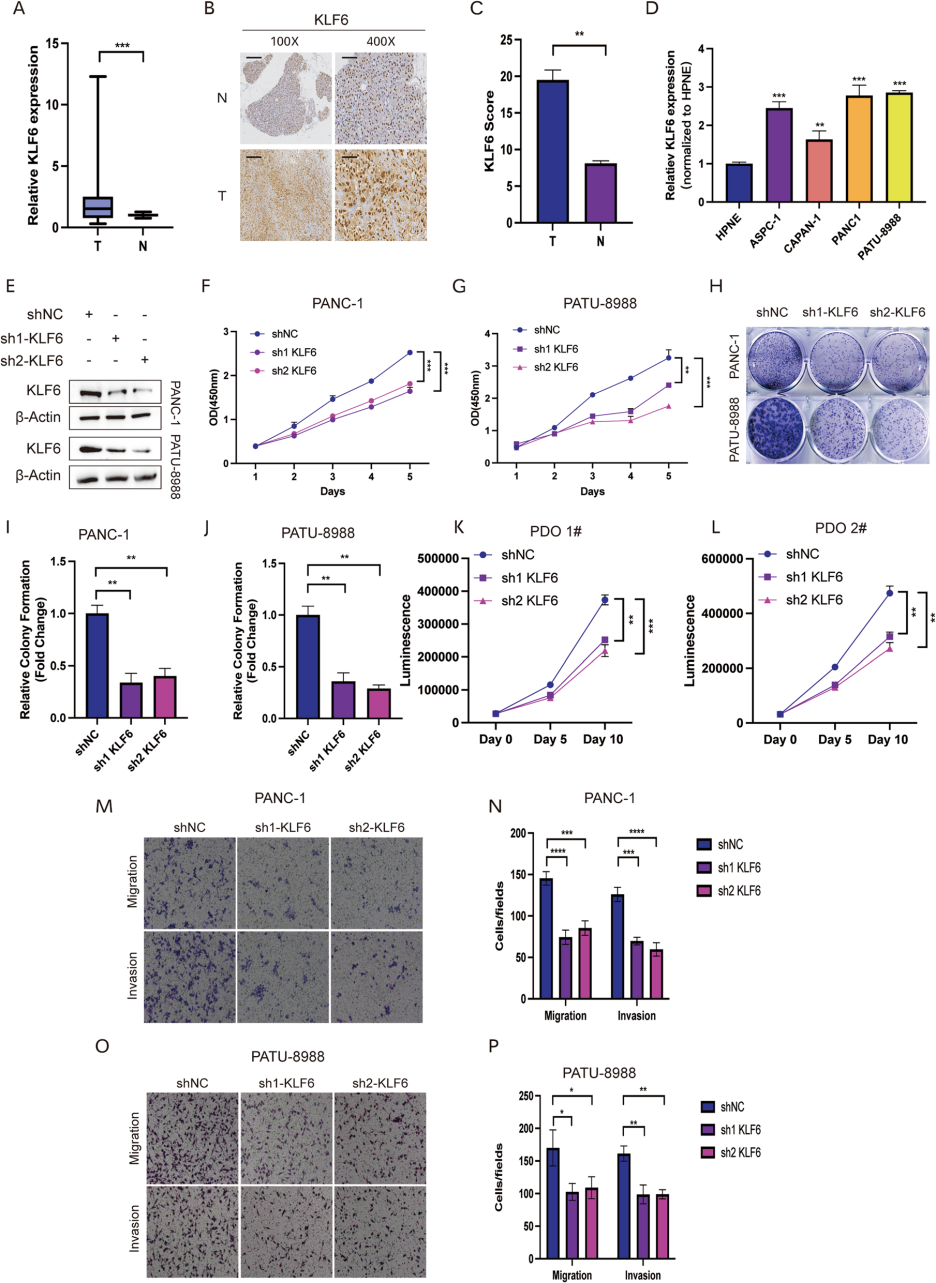
KLF6 promoted malignant progression of pancreatic cancer in vitro. **A** The expression of *KLF6* in tumor and adjacent tissues collected from 20 PDAC patients in Ruijin Hospital. **B** IHC staining images of KLF6 in clinical samples. **C** The IHC score revealed the infiltration of KLF6 in tumor and normal tissues. **D** Relative expression of *KLF6* in pancreatic cancer cell lines and normal epithelial cell line. **E** The protein levels of KLF6 after being knocked down in PATU-8988 and PANC1. **F**–**G** CCK-8 assays revealed the proliferative activity of KLF6 in PDAC. **H**–**J** Plate colony assays in PATU-8988 and PANC1 after transfection of shNC, sh1-*KLF6*, and sh2-*KLF6*. **K**–**L** The proliferative capacity of KLF6 in different patient-derived organoids on day 0, day 5, and day 10. **M**–**P** Transwell assays demonstrated the migration and invasion abilities of KLF6 in PDAC. (*p < 0.05; **p < 0.01; ***p < 0.001)

To validate the role of KLF6 in the progression of PDAC, we collected 20 paired tumor tissues and non-tumor tissues from Ruijin Hospital to examine the expression of *KLF6*. RT-qPCR analysis revealed that the expression level of *KLF6* was higher in tumor tissues and the IHC was further verified that KLF6 was infiltrated more in PDAC (Fig. 9A–C). In pancreatic cancer cells, *KLF6* was expressed higher in PATU-8988 and PANC-1 cell lines compared to others (Fig. 9D). Next, the pro-tumorigenic functions of KLF6 were explored. After knocking down *KLF6* in PATU-8988 and PANC1 cell lines, as expected, the proliferative activity was remarkedly suppressed (Fig. 9E–J). Moreover, similar results were observed in organoid proliferation (Fig. 9K, L and Figure S4A-D). Subsequently, the transwell assays determined that KLF6 enhanced the migratory and invasive abilities in pancreatic cancer cells (Fig. 9M–P). Above all, these results suggested that KLF6 may contribute to the progression of PDAC by promoting proliferation and metastasis activities.

### Pan-cancer analysis of KLF6

**Fig. 10:**
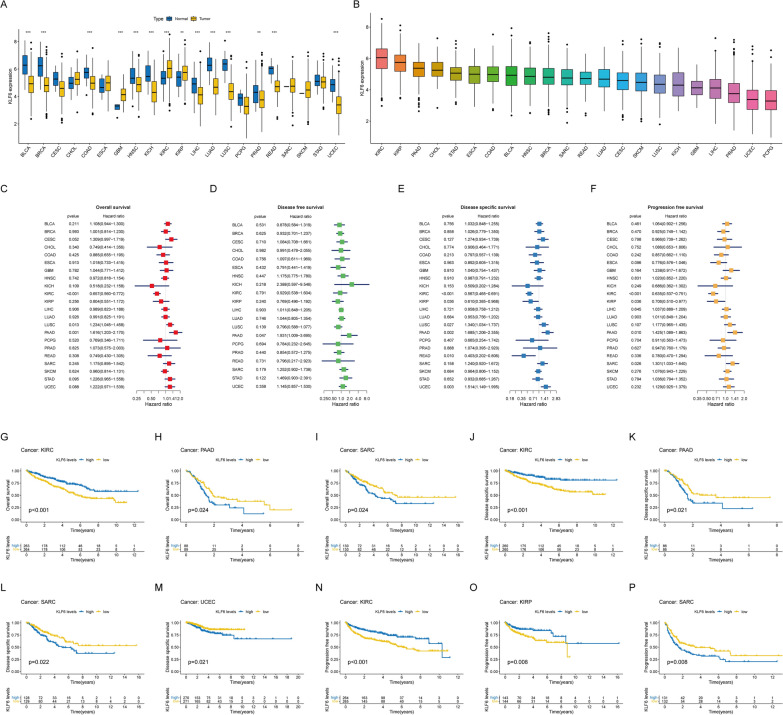
Analysis of *KLF6* in pan-cancer level. **A** The expression of *KLF6* in tumor and normal tissues of multiple cancers. **B** Relative expression of *KLF6* in pan-cancer. **C**–**P** The overall survival (OS), disease-free survival (DFS), disease-specific survival (DSS), and progression-free survival (PFS) analysis of *KLF6* in pan-cancer. (*p < 0.05; **p < 0.01; ***p < 0.001)

To investigate the potential value of *KLF6* in other tumors, pan-cancer analysis was performed. As shown in Fig. 10A, *KLF6* was significantly and differentially expressed across multiple tumor types. Moreover, *KLF6* was most highly expressed in kidney renal clear cell carcinoma tissues (Fig. 10B).

In addition, *KLF6* was closely related to overall survival, disease-free survival, disease-specific survival, and progression-free survival in pan-cancer (Fig. 10C–F). Further survival curves analysis confirmed it (Fig. 10G–P). Subsequently, we assessed the impact of *KLF6* in TIME of multiple cancers and found that the KLF6 was closely relevant to the immune microenvironment in a range of tumors (Fig. S5A-Q). Notably, *KLF6* is also associated with TMB and MSI in various types of cancer (Fig. S5R, S). Furthermore, as Fig. S5T, U demonstrated, KLF6 may act as a key factor in regulating the immune activity of pan-cancer.

## Discussion

Pancreatic cancer is a global health issue, characterized by rising incidence and mortality rates alongside limited clinical treatment options and low survival rates [26]. The need for novel biomarkers to facilitate early diagnosis and tailored therapies, and to improve prognosis remains critical and urgent.

KLFs, a family of 17 transcription factors with highly conserved zinc-finger structural domains, play crucial roles in tumor development, influencing proliferation, apoptosis, metastasis, and survival [6]. Despite understanding individual KLF genes’ impact on tumor progression, the potential interconnections within the KLF family in pancreatic cancer remain unexplored [27]. Consequently, this study aimed to explore the clinical value of whole KLF family genes in pancreatic cancer.

First, we investigated transcriptional differences and potential interactions among KLFs. Subsequently, according to differentially expressed KLFs, 178 patients with PDAC were classified into two distinct molecular phenotypes, which revealed significant differences both in the prognosis and clinicopathological characteristics. Enrichment analysis demonstrated that patients in cluster 1 exhibited increased activation of cancer-related pathways, including hypoxia, glycolysis, and the p53 pathway. TIME is infiltrated by a variety of immune cells, among which CD8 + or cytotoxic T lymphocytes exert tumor-killing function, while regulatory T cells attenuate effector T cell activity and promote immunosuppression in TIME [28, 29]. Different immune cell ratios could promote tumor growth and metastasis by shaping TIME and thus mediating the immune escape of tumor cells [30]. Cluster 1 showed a lower proportion of naive B cells infiltrated with CD8+ T cells. Furthermore, notable variations in stromal score, immune score, and estimate score were observed, indicating cluster 1 exhibited an immunosuppressive status and validating the prognostic differences between subgroups.

To enhance the clinical relevance of our study, we screened three genes (*KLF3*, *KLF5*, and *KLF6*) to construct the KLF_score prognostic model using Cox and LASSO regression analyses. KLF3 was considered a novel tumor-promoting transcription factor in colorectal cancer [31]. Specifically, highly expressed in ovarian cancer, KLF5 facilitated the process of ovarian cancer proliferation and metastasis and promoted PARP inhibitor resistance by remodeling the transcription of the key gene for homologous recombination, *RAD51* [32]. KLF6 was a tumor suppressor and inhibited the progression of a variety of tumors [33–35]. Based on the median KLF_score, patients were divided into high- and low-risk groups. The high-risk group displayed a worse prognosis than the low-risk group, along with heightened activity in cancer-related pathways such as glucose metabolism, p53, and the E2F pathway. Validation via K-M-plots and ROC curves of the validation cohort confirmed the robustness and reliability of the KLF_score signature. Additionally, immune checkpoint expression, immune pathway enrichment, TIDE scores, and TMB scores differed significantly between the high- and low-risk groups, suggesting higher immunological activation in the low-risk group, which aligns with their better overall survival. At the same time, it also meant that patients in the high-risk group were more suitable for immunotherapy to release their own immunosuppression and restore their immune activity, thus improving the anti-tumor effect.

Combination therapies usually work better than monotherapies, and this is especially true for cancer treatments based on checkpoint blockade with immunosuppression [36]. The drug sensitivity analysis could be better guidance in clinical treatment. Additionally, to screen for novel and potential targeted drugs, we utilized molecular docking and found three drugs that targeted *KLF3*, *KLF5*, and *KLF6*. These findings could provide valuable clinical relevance, guiding the choice of chemotherapy and targeted immunotherapy. Clinical characteristics impact prognosis. Therefore, we performed a multifactorial Cox analysis integrating clinical characteristics with the risk signature. Age, risk score, and N stage were independent prognostic factors in PDAC. The resultant graphic and calibration curves demonstrated the significant and efficient predictive ability of KLF score.

To further investigate the impact of key genes on tumor progression, we performed single-cell analysis and found *KLF6* was highly expressed in immune cells and strongly elevated in tumor cells. Then, we assessed the biological role of KLF6 in PDAC. KLF6 expression was highly elevated in pancreatic cancer samples compared to adjacent normal tissue samples. The proliferation and metastatic capacity were significantly suppressed by the downregulation of KLF6 in pancreatic cancer cells. Moreover, the clinical value of *KLF6* was assessed at the pan-cancer level.

Although we developed a KLF_score to serve as a reliable predictor of pancreatic cancer prognosis and demonstrated the contribution of KLF6 to the development of the disease, our study has some limitations. First, the dataset employment had a small sample size. In some cases, the clinical characteristics of those patients were incomplete. Future validation using larger datasets from multiple sites is needed. Second, the mechanisms underlying the impact of KLF6 on pancreatic cancer progression are still unknown. Moreover, single-cell sequencing data revealed that *KLF6* was strongly expressed in tumor immune cells, such as macrophages, indicating that KLF6 may participate in remodeling TIME, enhancing the progression of pancreatic cancer. These challenges warrant future exploration.

## Conclusion

In general, our study illustrated the clinical relevance and significance, tumor microenvironment characteristics, and immune infiltration features of the KLF family in pancreatic cancer. In addition, a reliable and accurate KLF gene-based prognostic model was presented. Further cellular analysis on KLF6 confirmed the role it plays in the malignancy of pancreatic cancer. These findings might be useful for early diagnosis and targeted treatment on pancreatic cancer.

### Supplementary Information

Supplementary material 1: Figure S1. NMF clustering applied on differently expressed KLFs. (A) Nonnegative matrix factorization rank survey. (B) Consensus matrix heatmaps for k=2-10.

Supplementary material 2: Figure S2. Enrichment analysis of high- and low-risk group. (A) GSVA analysis indicated the different biological pathways of high- and low-risk groups. The yellow and blue respectively represented activated and suppressive pathways. (B-G) GSEA displayed enriched gene sets in patients with high- and low-risk score groups.

Supplementary material 3: Figure S3. Drug sensitivity analysis. (A-L) Difference IC50 of axitinib, camptothecin, gefitinib, JNK.Inhibitor.VIII, nilotinib, pazopanib, salubrinal, sunitinib, bleomycin, lapatinib, paclitaxel, and vinorelbine in high- and low-risk groups. (* *p* < 0.05; ** *p* < 0.01; *** *p* < 0.001).

Supplementary material 4: Figure S4. KLF6 inhibited the proliferation of patient-derived organoids from PDAC. (A-B) Representative pictures of PDO 1# (A) and PDO 2# (B) on day 0, day 5, and day 10 after transfected shNC, sh1 *KLF6* and sh2 *KLF6*. (C-D) The area size was compared on day 0, day 5, and day 10 respectively in shNC, sh1 *KLF6*, and sh2 *KLF6 *groups. (* *p* < 0.05; ** *p* < 0.01; *** *p* < 0.001).

Supplementary material 5: Figure S5. Relevance of *KLF6* and tumor immune microenvironment in pan-cancer. (A-Q) The correlation of stromal score and *KLF6* expression, estimate score and *KLF6* expression, immune score, and *KLF6 *expression in pan-cancer. (R) Tumor mutation burden of *KLF6* in pan-cancer. (S) Microsatellite instability of *KLF6* in pan-cancer. (U) The co-expression analysis of immunostimulators and *KLF6* in pan-cancer. (V) The co-expression analysis of immunoinhibitors and *KLF6 *in pan-cancer. (* *p* < 0.05; ** *p* < 0.01; *** *p* < 0.001).

Supplementary material 6: Table S1. Univariate Cox regression analysis.

Supplementary material 7: Table S2. Multivariate Cox regression analysis.

## Data Availability

The datasets presented in this study can be found in the UCSC Xena and GEO databases.

## Abbreviations

PDAC: Pancreatic ductal adenocarcinoma
KLFs: Krüppel-like factors
NMF: Non-negative matrix factorization
TIME: Tumor immune microenvironment
LASSO: Least absolute shrinkage and selection operator
scRNA-seq: Single-cell RNA sequencing
CNV: Copy number variation
DEGs: Differentially expressed genes
GSVA: Gene set variation analysis
GSEA: Gene set enrichment analysis
ROC: Receiver operating characteristic
PCA: Principal component analysis
TMB: Tumor mutation burden
MSI: Microsatellite instability
AUC: Area under the ROC curve
K-M: Kaplan–Meier
PDO: Patient-derived organoid
ETCs: Epithelial tumor cells
