# Supplementary figures and images for: Comprehensive analysis of KLF family reveals KLF6 as a promising prognostic and immune biomarker in pancreatic ductal adenocarcinoma
Source: Cancer Cell Int. 2024 May 21;24:177. doi: 10.1186/s12935-024-03369-3 (PMC11106939; doi:10.1186/s12935-024-03369-3)

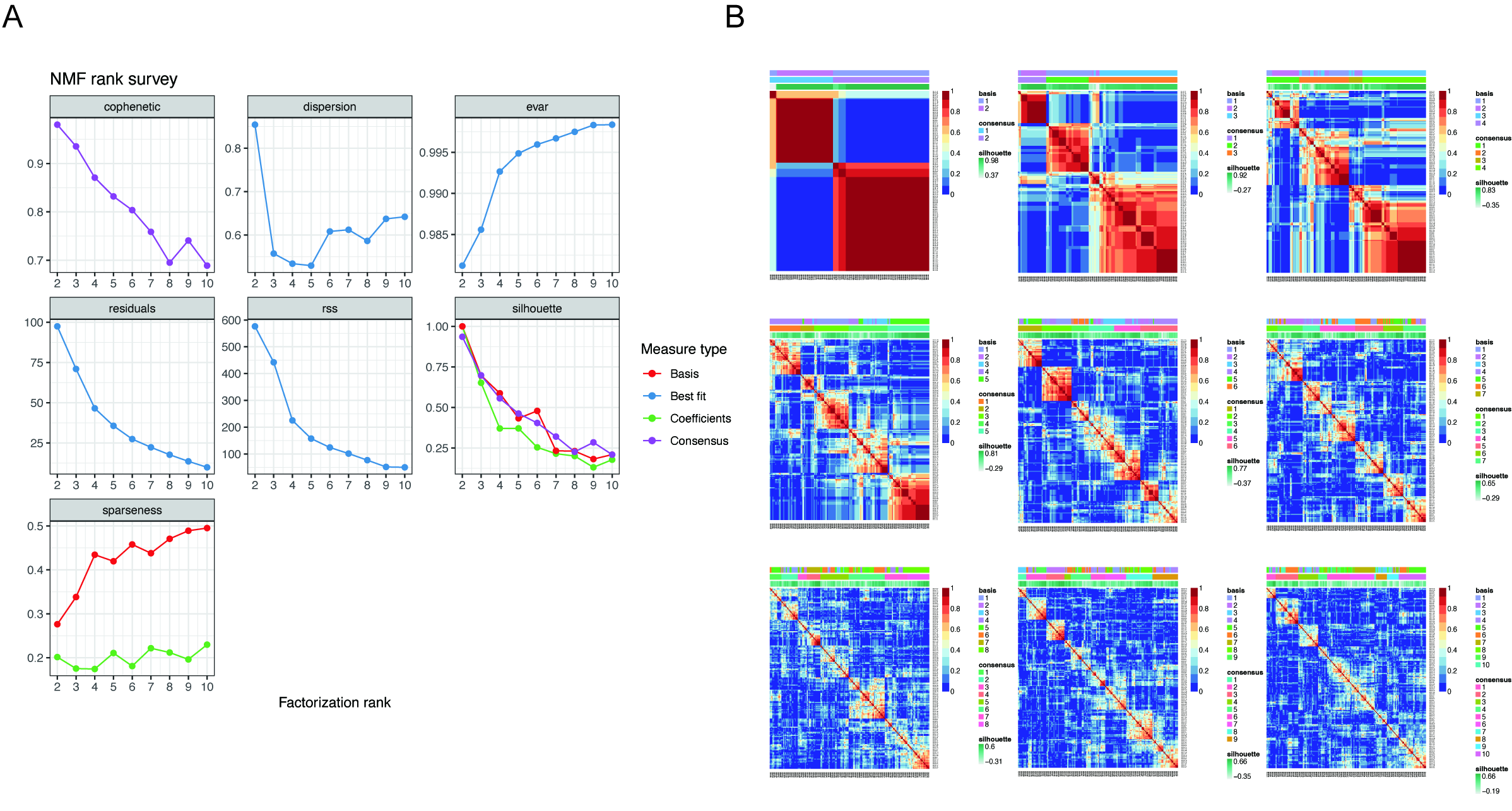

Supplement: Supplementary file 1 — Supplementary material 1: Figure S1. NMF clustering applied on differently expressed KLFs. (A) Nonnegative matrix factorization rank survey. (B) Consensus matrix heatmaps for k=2-10. [file 12935_2024_3369_MOESM1_ESM.tif]

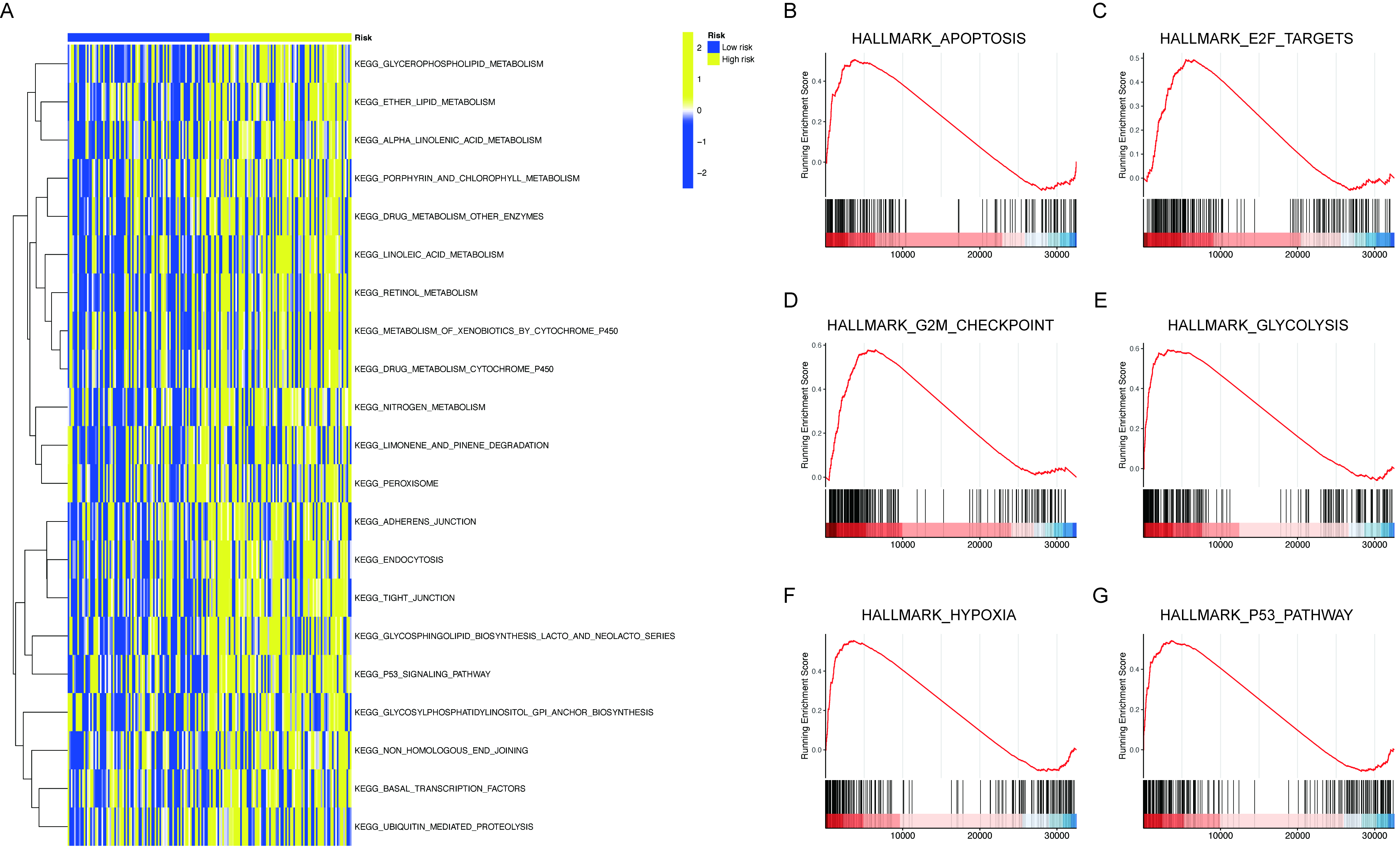

Supplement: Supplementary file 2 — Supplementary material 2: Figure S2. Enrichment analysis of high- and low-risk group. (A) GSVA analysis indicated the different biological pathways of high- and low-risk groups. The yellow and blue respectively represented activated and suppressive pathways. (B-G) GSEA displayed enriched gene sets in patients with high- and low-risk score groups. [file 12935_2024_3369_MOESM2_ESM.tif]

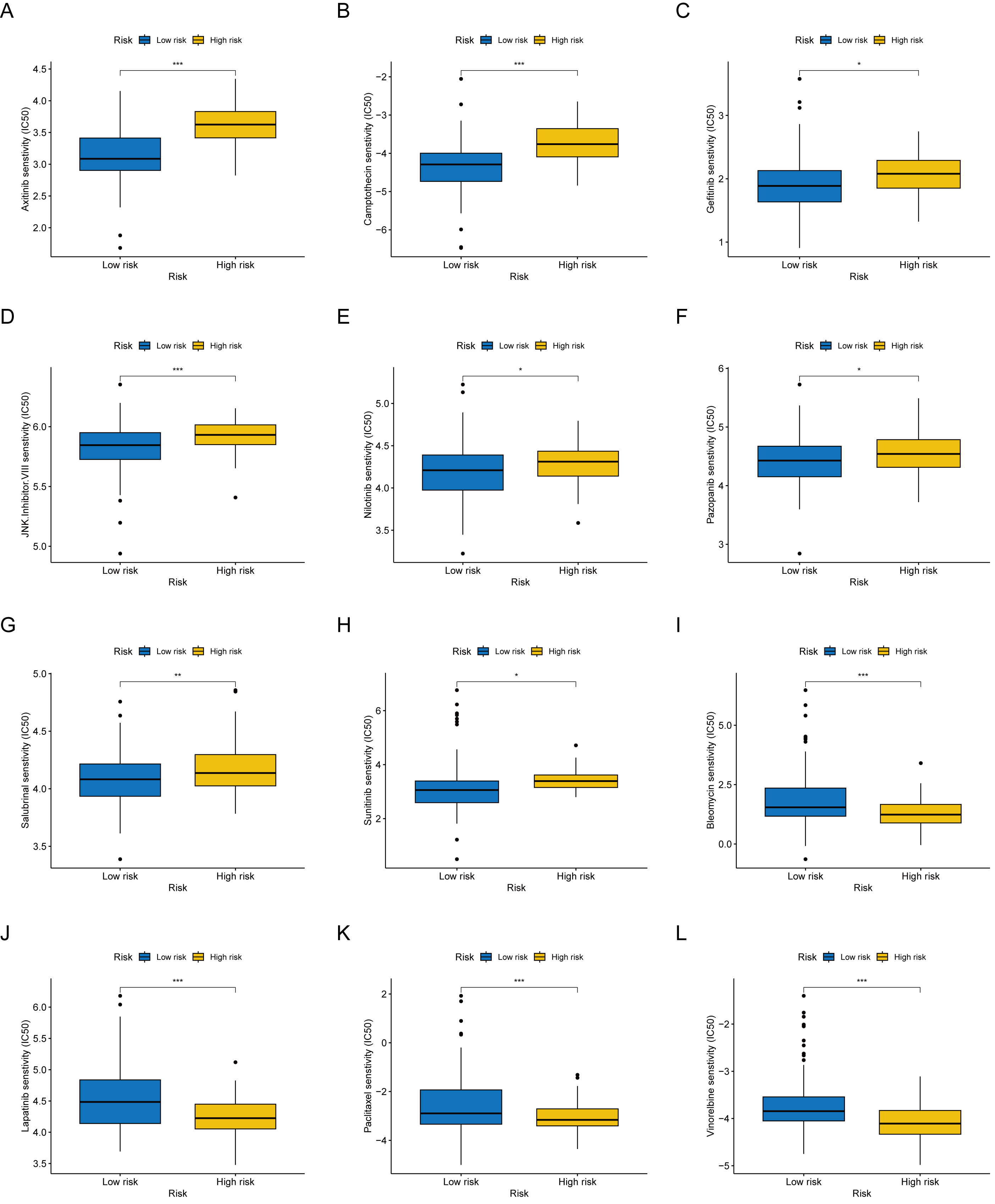

Supplement: Supplementary file 3 — Supplementary material 3: Figure S3. Drug sensitivity analysis. (A-L) Difference IC50 of axitinib, camptothecin, gefitinib, JNK.Inhibitor.VIII, nilotinib, pazopanib, salubrinal, sunitinib, bleomycin, lapatinib, paclitaxel, and vinorelbine in high- and low-risk groups. (* p < 0.05; ** p < 0.01; *** p < 0.001). [file 12935_2024_3369_MOESM3_ESM.tif]

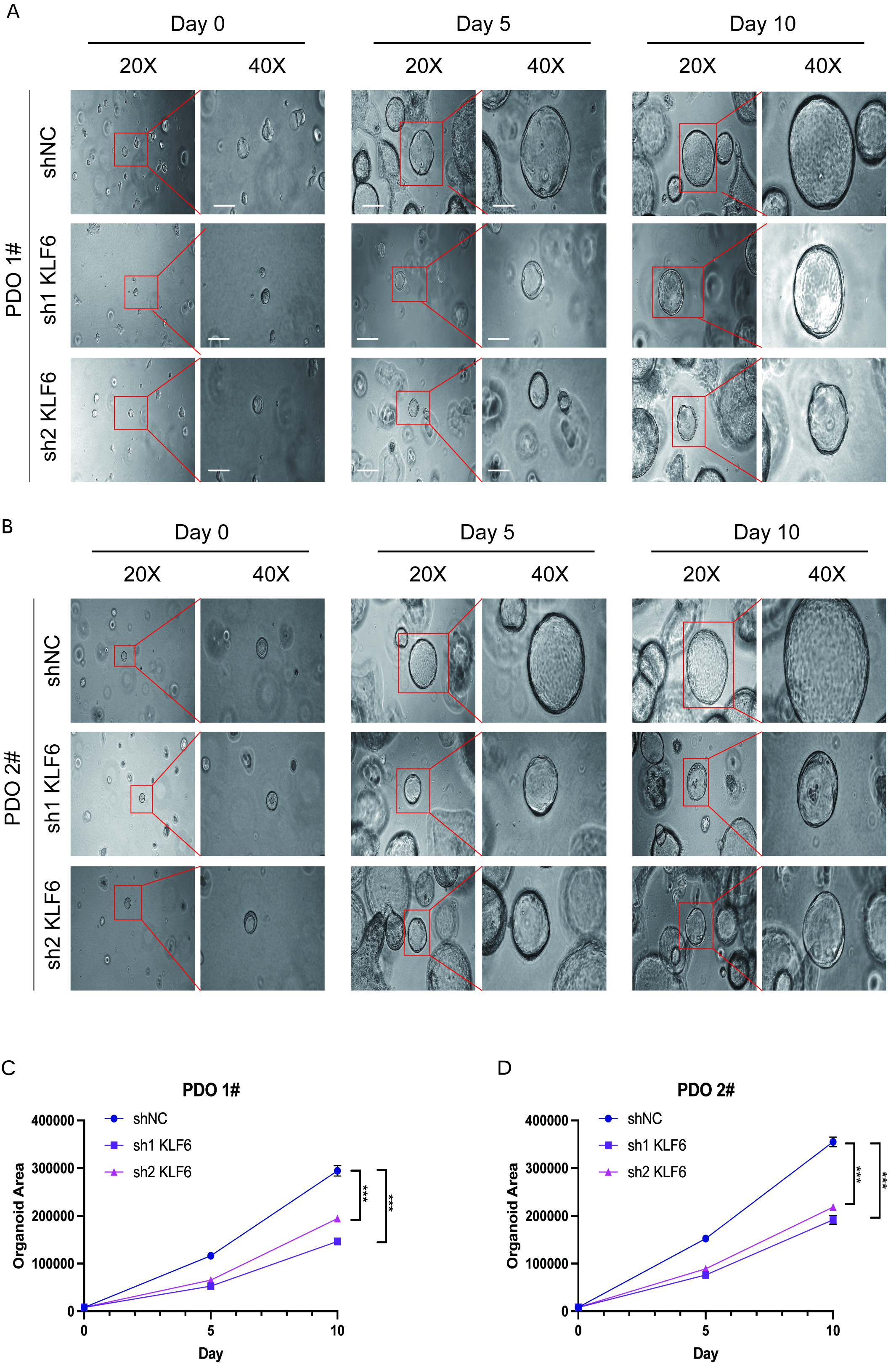

Supplement: Supplementary file 4 — Supplementary material 4: Figure S4. KLF6 inhibited the proliferation of patient-derived organoids from PDAC. (A-B) Representative pictures of PDO 1# (A) and PDO 2# (B) on day 0, day 5, and day 10 after transfected shNC, sh1 KLF6 and sh2 KLF6. (C-D) The area size was compared on day 0, day 5, and day 10 respectively in shNC, sh1 KLF6, and sh2 KLF6 groups. (* p < 0.05; ** p < 0.01; *** p < 0.001). [file 12935_2024_3369_MOESM4_ESM.tif]
